# Q&A: Why use synchrotron x-ray tomography for multi-scale connectome mapping?

**DOI:** 10.1186/s12915-017-0461-8

**Published:** 2017-12-21

**Authors:** Yeukuang Hwu, Giorgio Margaritondo, Ann-Shyn Chiang

**Affiliations:** 1 Institute of Physics, Academia Sinica, Nankang, Taipei 11529 Taiwan; 2 Ecole Polytechnique Fédérale de Lausanne, Lausanne, CH 1015 Switzerland; 3 Brain Research Center, National Tsing Hua University, Hsinchu, 30013 Taiwan

## Abstract

To understand how information flows and is used in the human brain, we must map neural structures at all levels, providing visualizations similar to those of Google Earth for continents, countries, cities, and streets. Unfortunately, the imaging and processing techniques currently used in connectomics projects cannot achieve complete mapping for the brains of large animals within the timespan of a typical research career. However, feasible improvements in x-ray imaging would change this situation. This Q&A discusses synchrotron x-ray tomography, an exciting new approach for in situ mapping of whole-brain wiring diagrams at multiple levels of spatial resolution.

## In a Presidential Lecture at the 2016 Annual Meeting of Society for Neuroscience, you presented the use of synchrotron x-ray tomography for mapping whole body connectomes in *Drosophila*. Why do we need x-ray imaging for connectomics?

Recent advances in imaging technologies open the door to mapping a complete wiring diagram of the human brain, a long-standing goal in neuroscience. However, each of the imaging techniques being currently deployed provides different levels of visualization and can give only a partial picture: medical-type imaging reveals connectivity between brain regions; visible light microscopy detects neuronal circuits at the cellular level; and electron microscopy (EM) identifies synapses and intracellular structures. In theory, a complete connectome for a whole brain, including all cells and their synaptic connections, could be achieved by a three-dimensional reconstruction of EM images. But in practice the time required limits this approach to mapping a small part of the human connectome at least for the foreseeable future.

We sought an alternative to these techniques on deciding that we wanted to map the whole body connectome of *Drosophila* in order to understand the neural basis of its behavior. X-ray microscopy offered advantages in speed and resolution made more apparent and exploitable since its development using modern synchrotron facilities [1, 2]. To meet the challenge of connectome mapping, however, certain performance improvements were required, notably to handle large specimens—the entire animal brain and body—with sufficient image contrast and tunable resolution to detect the fine connections. Overcoming these obstacles took more than a decade, but as a result x-ray imaging is now, in our view, the technique of choice for whole-body connectome mapping.

Many of the advanced x-ray imaging techniques of today are based on synchrotron sources. Synchrotron x-rays offer high brightness with deep-penetration for in situ visualization at a high speed of internal structures within a large tissue—similar to medical computer tomography, but with much better spatial resolution for obtaining multi-level views, ranging from a large brain region to a single synapse. Importantly, the in situ structures mapped by x-rays can be accurately aligned with respect to in situ functional data taken with confocal and two-photon microscopies. Combining x-ray and optical microscopy in situ data will allow the combination of knowledge derived from different laboratories for a full understanding of how the brain neurons respond to a sensory input and control the body to implement the corresponding behavior.

**Fig. 1:**
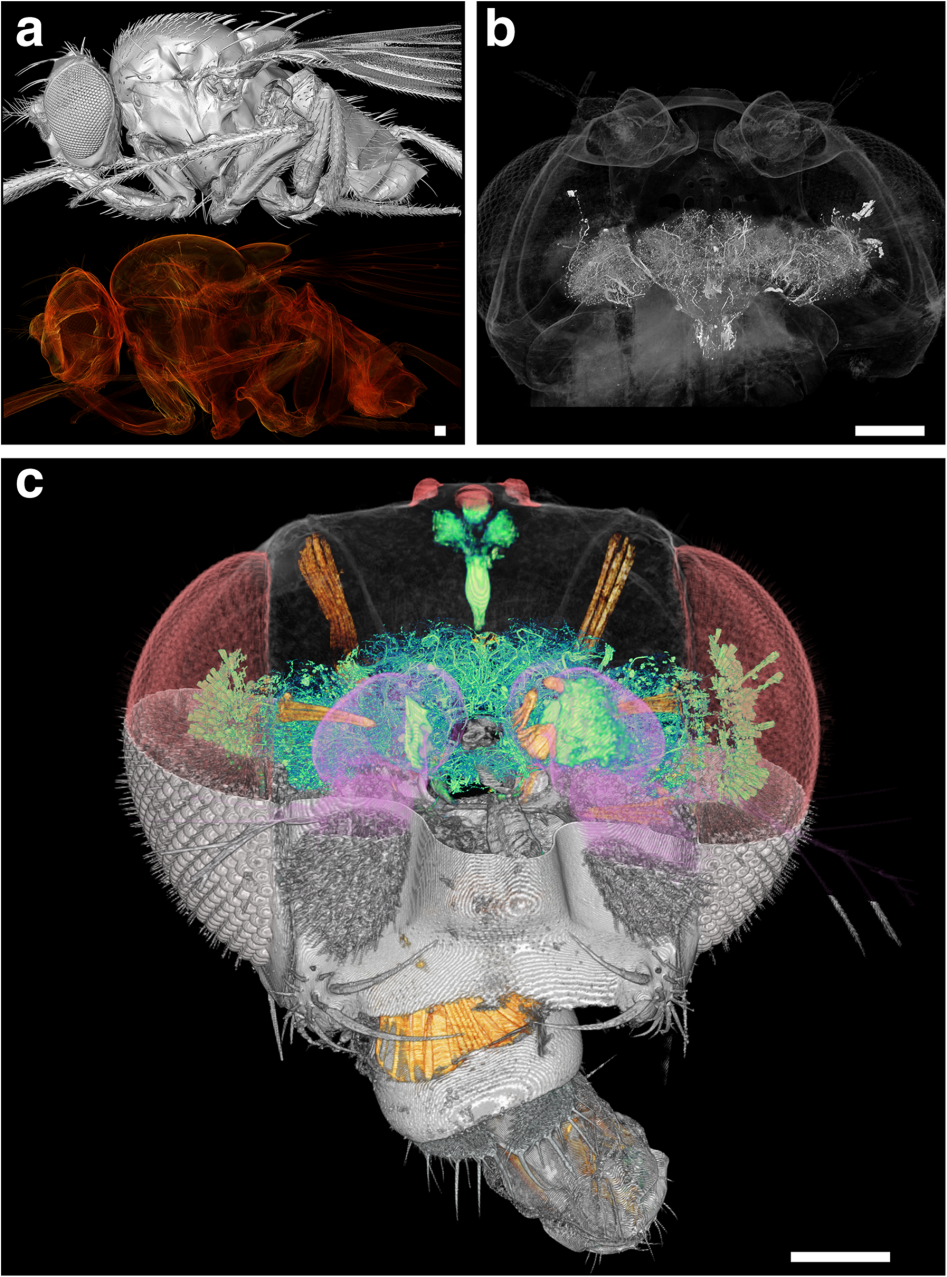
Tomographic 3D reconstruction of the whole-body *Drosophila.* **a** Surface (*top*) and volume (*bottom*) rendering of the whole body. Without fixation and staining, enhanced phase contrast can clearly reveal the skeleton but not the brain. **b**, **c** The head of a *Drosophila* treated by Golgi-staining. The top half of the skull in **c** is made transparent to show the brain and the neurons inside. *Scale bar* 200 μm

As a proof of concept, we have imaged with synchrotron x-ray tomography not only the whole *Drosophila* brain and body (Fig. 1) but also the whole mouse brain. Based on the imaging results of more than 500 *Drosophila* and tens of mouse brains, we believe that synchrotron x-ray tomography is an ideal tool for achieving a three-dimensional (3D) reconstruction of the whole human brain in the not-too-distant future.

## How does x-ray imaging work and what are its advantages for connectome mapping?

X-ray imaging was the very first use of x-rays, inaugurated by Roentgen a few hours after his discovery. The technology transfer to medical radiology was one of the most rapid ever and x-ray imaging remains today, by far, the main field of medical imaging activity. The working principle of the x-ray imaging we use for brain imaging is no different from that used in common medical radiology. An x-ray source illuminates the object and an imaging device detects the x-rays passing through, forming shadow images revealing the inside structure of the object. X-ray imaging emerges as the top candidate for brain mapping because of its excellent performance and unique characteristics:

1. High penetration: in most of the x-ray wavelength range, the absorption by materials is very weak, so the radiation reaches deeply into the specimens as required for 3D imaging, in turn essential for connectome mapping.
2. 3D imaging: this is realized with powerful specialized approaches for tomographic reconstruction—invented for medical use but now expanding into many other applications.
3. Spectroscopy: the interactions of x-rays with materials are mostly linked to electron core levels, whose energies are determined by the elements in the specimen and by their chemical status. Many x-ray spectroscopy techniques—such as x-ray fluorescence—exploit this fact to reveal the chemical composition and properties. In many cases, these techniques can be implemented with high spatial resolution, becoming “spectromicroscopies”. This capability could contribute in the future to the connectome mapping projects by adding microscopic chemical information to the structural information.
4. Short wavelengths: an advantage since the resolution is often determined by the “diffraction limit”, which causes a linear relationship between resolution and wavelength to the advantage of short-wavelength (≤ 1 Å) x-rays.

## Resolution: what level do we need for whole-brain imaging to map the connectome?

An imaging technique for this task must provide sufficient spatial resolution to reliably detect individual neurons and their major connections. Considering the size of dendritic connections and synapses, this means a nanoscale resolution (<100 nm). Such a high resolution increases the image-taking time, making the mapping of the entire brain unrealistically long. A sound connectome mapping strategy must then combine different imaging techniques with different resolution levels, using very high resolution only when strictly needed.

Since the neural network densely occupies the whole brain, the required resolution must be achieved in all directions. The level of resolution could be different in different directions (for example, in two-dimensional (2D) electron microscopies)—but isotropic resolution is preferable for 3D imaging because of the straightforward analysis of the data.

For a whole-brain mapping strategy, besides resolution one must consider the field of view (FOV). A small FOV would jeopardize the advantages of high resolution by increasing the number of images to be taken and the total time for whole-brain mapping. The FOV-to-resolution ratio must thus be as large as possible.

**Fig. 2:**
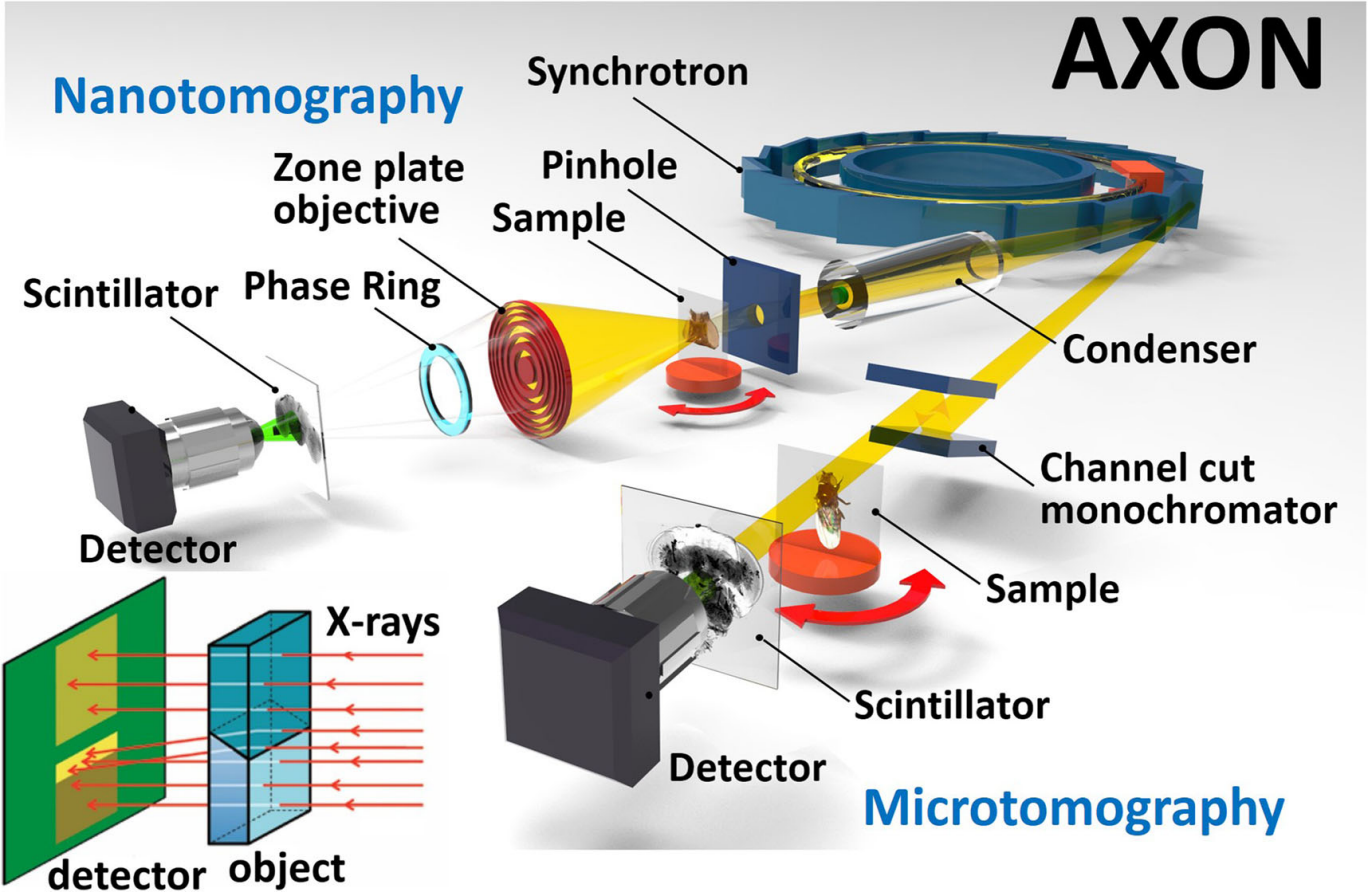
The AXON system with two types of beamlines used in this study. On the *left*, a nanoresolution transmission x-ray microscopy beamline, such as the NSRRC (National Synchrotron Radiation Research Center, Taiwan) TLS 1B and PLS-II (Pohang Light Source, Korea) 7C facilities, which achieve < 20 nm resolution [1] and, on the *right*, a microtomography beamline, such as the NSRRC TLS 1A and TPS U23, and the PLS-II 6C facilities. The *inset* (*lower left*) illustrates the edge enhancement effect due to phase contrast from a boundary between two regions of different refractive index

X-ray imaging satisfies all of the above requirements [3–5]. Our new methodology, Accelerated X-ray Observation of Neurons (AXON), combining synchrotron x-ray microtomography and optimized staining, was specifically designed with performances suitable for mapping individual neurons in the whole brains of animals used as model organisms. By projecting x-rays through a sample onto a scintillator and then capturing the visible light images with a high resolution optical microscope, AXON microtomography (micro-AXON) allows us to image the interior of a whole fly head at < 0.5 μm resolution. With similar procedures, the AXON nanotomography (nano-AXON) produces ~ 20 nm resolution, by magnifying the projected image with a Fresnel zone plate, and enhancing the contrast with a Zernike type phase ring as previously described [6]. To overcome the limitation of the field of view, we combined multiple projection images for neuron fibers extending over a large volume. This technique, however, works best by zooming in the region of interest, identified by micro-AXON. Imaging was performed in one facility, as shown in Fig. 2, at two resolution levels: micro-AXON at < 0.5 μm resolution for single neurons and their major connections within large brain volumes, and nano-AXON at ~ 15 nm resolution for sub-cellular structures and fine connections. In this flexible strategy, using the same photon source for different resolution levels is very advantageous.

The 3D information carried by the 2D images can be extracted by tomographic processing [7]. This typically requires large sets of 2D images for different specimen-detector geometries, obtained by rotating the sample or the detector. Specialized computer programs convert such images into 3D volumetric data without deteriorating the resolution.

## Contrast: does x-ray imaging of the brain produce enough contrast to image neurons and their connections for connectome mapping?

The answer is negative for unprocessed brain specimens, but becomes positive after adequate staining. X-rays are characterized by a weak absorption that, as we saw, allows high penetration. But low absorption also means low contrast. This must be corrected with suitable staining/labeling techniques, increasing the x-ray absorption of relevant features including neurons and connections.

Unfortunately, common labeling agents for biomedical imaging—such as the fluorescent fluorophores—do not work for x-rays. One needs instead staining agents that are strong x-ray absorbers, such as those containing heavy metals.

An alternative to staining is offered by x-ray imaging contrast based on the changes in the wave phase rather than on absorption [8]. The phase of a wave propagating through a specimen can be modulated differently by the refractive index in different regions. One notable example is the refraction observed for visible light at a water–air interface. In the case of x-rays, with an adequate positioning of the detector and the specimen, phase contrast can produce fringes that dramatically enhance the visibility of boundaries between different parts of the specimen (inset of Fig. 2). Phase contrast offers multiple advantages over absorption contrast. In fact, x-ray absorption decreases with the atomic number and exhibits only small differences between most materials in bio-systems. The differences are larger for refractive index, which determines phase contrast. In practical tests, phase contrast has enabled x-rays to image isolated cells [1].

Could this mechanism solve the contrast problem in whole-brain connectome mapping? Unfortunately, no. Phase contrast tomography works very well for many internal body structures, such as endoskeleton and muscles [9]. But the phase differences between neurons and the extracellular matrix are minimal, so phase contrast is not very useful for directly mapping neural connections. However, it is quite important for connectome mapping by producing high contrast for all the different parts of the animal heads. These clear 3D features, such as the clearly visible components of the muscle and exoskeleton in Fig. 1, can be used for aligning 3D brain images from different specimens.

## Speed: why is it that only x-ray imaging offers the overall performance required for whole-brain mapping?

A key issue in planning a whole-brain mapping strategy is the total time required. The total time for mapping is determined by the number of required raw images and the time to take and to computer-process each image. The specimen volume covered by each single image with the required resolution determines the number of image pixels and consequently the number of needed projection images. Due to the high penetration of x-rays, this specimen volume is only influenced by the spatial resolution level and by the FOV.

The ratio of the FOV to the image pixel is determined by the technical characteristics of the image detector and can be tuned by the magnification factor (achieved, for example, with x-ray lenses). Working at 1 μm^3^ resolution, a synchrotron provides the x-ray flux sufficient to collect a projection-image set for tomography of a 10^9^ μm^3^ volume within a second [10]. Roughly speaking, this corresponds to a total image-taking time of ~ 5 × 10^2^ s for a whole mouse brain and of ~ 10^6^ s (~12 days) for the human brain. These times are, however, unrealistic and achievable only by fully optimizing all the involved techniques. More realistic projected performance falls below these extreme levels. Even so, the evaluated total image-taking time of months or years would be acceptable. And no other imaging technique gets even close to this performance, their image-taking times being longer by orders-of-magnitude.

In essence, the 3D nature of x-ray imaging makes it ideal for complex 3D structures, and therefore superior to any other imaging technique in terms of speed, the crucial obstacle along the path to whole-brain connectome mapping.

## Synchrotrons: why use them? Does their coherence matter?

Synchrotrons are truly superior x-ray sources for imaging applications [2]. In order to fully appreciate their role, one should consider several characteristics: flux, brightness and coherence.

1. The high emitted flux *F* of a synchrotron source increases the signal-to-noise ratio, shortening the image-taking time. This is certainly important, but does not give a complete picture.
2. Brightness: focusing x-rays—as required for many microimaging techniques—is facilitated if the source is small and with a narrow angular range. In fact, the product of the beam transverse section and of its two-dimensional angular spread is conserved along an optical system. In practice, strong focusing requires large optical devices that are technically challenging and expensive—unless the source size *Σ* and (two-dimensional) angular spread *Ω* are small. The brightness (or brilliance) *B* = constant × *Φ*/*(ΣΩ)* combines these properties: a good source has a high brightness.
3. Coherence is the property that enables the emitted radiation to produce wave-like phenomena like interference and diffraction, and also phase contrast. There are two types of coherence: “spatial” (or “lateral”) and “time” (or “longitudinal”). Time coherence, related to the emitted wavelength bandwidth Δ*λ*, is measured by the “coherence length” *L*_c_ = *λ* ^2^/Δ*λ*. Spatial coherence is measured by the “coherent power”, a parameter proportional to Δ*λ* ^2^/(*ΣΩ*). Synchrotron x-ray sources produce high coherence for phase contrast imaging.

These parameters can be understood by considering, for example, diffraction by a pinhole. A source with small size *Σ* and an infinitely narrow bandwidth Δ*λ* always produces a detectable diffraction pattern. But if Δ*λ* becomes large, different wavelengths produce different diffraction patterns, whose superposition may no longer exhibit diffraction-revealing fringes. Likewise, different points in a large-size source produce different patterns whose superposition may not exhibit fringes.

The above values characterize x-ray sources in terms of imaging performance, such as contrast and spatial resolution. Synchrotron x-rays reach better values than any other x-ray source by orders of magnitude. For example, the spatial resolution we achieved with synchrotron microtomography (<0.5 μm) is difficult to obtain with other xray sources, due to insufficient flux, brightness or coherence. Thus, in our view, synchrotron x-rays remain the ideal choice for superior image performances.

## Staining: sparse or complete? Advantages and limitations of different strategies

As mentioned earlier, for connectome mapping the low x-ray contrast must be compensated for by staining the neurons with high x-ray-contrast materials. Specifically, small particles—typically metallic—delineate the relevant microstructures if they “decorate” them: structure-specific staining is essential.

Staining methods can be imported from other imaging techniques but must be optimized for connectome mapping. We want to image as many neurons as possible: could we simply stain all neurons? Unfortunately, no: complete staining does not work for the brain, whose volume is mostly filled by neurons and connections; tomographic processing cannot be performed if the x-ray transmission is excessively blocked—unless a nanometer 3D resolution is used to separate all connected cells. Nanotomography offers this resolution, but image taking is very long.

We solved this problem by going back to the original approach by Golgi and Cajal to sparsely stain neurons with heavy metals, a popular neuroimaging method for 2D visible-light microscopy. For a century, the Golgi method, with its characteristic “random” staining, enabled many generations of neurobiologist to capture the complete structure of individual neurons in extremely packed brains. As we recently demonstrated (Fig. 1), this method works even better for x-rays, revealing with sub-micron resolution individual neurons delineated by the metal stain [6].

The Golgi method stains a neuron either completely or not at all, depending on a process that is not completely understood, by which the heavy metal ions diffuse inside of the cell body and are reduced and nucleated. The number of stained neurons is low, typically 1–5% [11, 12] in mouse brains. This allows us to use a plan based on sparse staining and on a statistical approach (similar to that used for human genome mapping), morphing and fusing many different partial 3D images to cover all neurons and connections. With an estimated 1–5% Golgi staining coverage of *Drosophila* brains, this will require partial images of > 100 whole brains. We have already acquired that many images for *Drosophila*, and we are completing the same strategy for mouse brains.

The staining coverage can be increased by increasing the staining time, or by other modifications of the procedure. From our *Drosophila* and mouse tests, we estimate that the Golgi-staining coverage could be expanded by > 30% before resolving the connected structures becomes problematic. This increase would reduce the number of brains required for complete connectome mapping—something very beneficial for mouse and human brains due to the limited specimen availability.

## Specimen preparation: how to guarantee the required isotropic 3D resolution? Can x-rays perform live imaging with no specimen preparation?

Besides staining, other aspects of specimen preparation are critical in high-resolution x-ray tomography. The reconstruction from raw images often yields poor resolution or fails completely due to specimen instability, specifically local distortion due to heating, dehydration or other types of radiation damage. Indeed, the standard “filtered back projection (FBP)” reconstruction, an efficient way to convert 2D images taken at different viewing angles with respect to the x-ray beam into 3D volume data [13], does not tolerate any of the specimen parts corresponding to the minimum reconstruction volume (voxel) to deviate from its ideal circular trajectory by significantly more than its own size.

This imposes a stringent condition to specimen stability: radiation damage and other causes of distortion must be minimized. Note that several iterative reconstruction algorithms are designed to correct minor distortions but they also prolong the reconstruction time and thus slow down the entire process.

It is thus very important to prepare the specimens so that they are sufficiently stable under the effects of radiation, heating or other factors during image acquisition. In our experiments, we extensively tested standard fixation methods and successfully used the fixation method described in [6] to meet the requirements of connectome mapping.

As to the possibility of x-ray imaging of live specimens, this is of course routinely achieved by medical radiology. And x-ray microtomography has demonstrated excellent live-imaging capabilities in a study of vasculature caused by tumor microangiogensis in mice [14]. In this case 3D imaging of even the smallest capillaries was achieved with the aid of contrast agents administered through the vessels. One cannot, however, apply contrast or labeling agents to similarly delineate neurons in live brains, so live-specimen imaging is not an option for connectome mapping.

## Reconstruction: what algorithms can be used and what is their level of performance?

In essence, tomography reconstruction first converts a series of 2D images into quantitative values of the absorption coefficient for each specimen voxel, which are then used to produce 3D maps by a procedure called volume rendering [15]. With the most effective FBP method [13], a typical reconstruction can be completed within a few minutes on a standard computer workstation.

There is certainly room for improving the reconstruction performance further to accelerate connectome mapping and several new methods have been proposed to exploit increasing computer power. A common strategy is to obtain an accurate 3D structure by iteratively correcting at each step the results of the previous step with fits of 2D images. This approach, when it converges, can reduce the number of required 2D images and/or their angular range [16].

Artificial intelligence is likely to play a key role in the reconstruction process [17]. We specifically envision using the a priori knowledge of individual neuron shapes and of the connection distribution and characteristics to develop more effective reconstruction algorithms. This could notably accelerate the convergence of iterative reconstruction and achieve the best compromise between final image quality and overall speed.

## Image processing: how are the raw images transformed into structural data?

A successful tomography reconstruction should preserve all the details of the network structures down to the targeted 3D resolution, producing accurate “bit map” voxel-by-voxel volumetric data. The “bit map” information is then converted to a vector representation of the network. In connectome mapping, the critical information for each voxel is if it belongs or not to the neuron network. Therefore, the conversion can be limited to “black-and-white” maps.

This is achieved with the “segmentation” process. When visually inspecting the final tomographically reconstructed slices, one can recognize if a voxel is stained or not by judging the grey scale value of the voxels. If the value, the local x-ray absorption coefficient, is higher than a well-selected threshold corresponding to the unstained tissue absorption, this indicates that the voxel includes absorbing components other than the tissue. One can thus conclude that it is affected by the heavy-metal Golgi staining and must, therefore, be part of the neural network. The final steps of image processing must connect all the voxels that are related to one specific neuron and provide a picture of the complete structure.

This procedure is not trivial. Noise interferes with it, in particular when the signal is weak: one needs special algorithms [18] to handle the weak contrast of very fine structures. But the process was optimized and is now fast enough to be part of the image processing procedure without causing excessive delays.

**Fig. 3:**
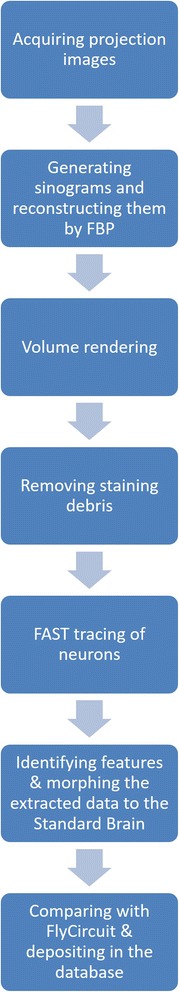
The tomographic reconstruction procedure for constructing a database from x-ray micro-radiography images

The segmentation and neuron tracing steps produce a vector representation of the network that does not occupy too much memory space—and can be easily used for further structural analysis without accessing the original images. Figure 3 shows the standard procedures for the image processing described above.

## How do you obtain a complete connectome from different brains with structural variations?

Brains are inherently plastic. In *Drosophila*, an adult brain is subdivided into ~ 50 small regions, called local processing units [19]. Most units are well separated from each other with distinct boundaries as a result of clustered neurites from its intrinsic local interneurons mixed with axons/dendrites from extrinsic input/output neurons. At the mesoscopic level, information from each sensory modality is relayed by stereotyped projection neurons and processed by specific sets of local processing units [20]. Thus, regardless of local synaptic plasticity, a neuron hardly changes its pre- and post-synaptic partners in a well-developed brain.

Our high-speed microscale x-ray mapping gives us an opportunity to address neuron-to-neuron connectivity in the whole *Drosophila* brain by a statistical approach. With a large number of datasets, > 500 brains of *Drosophila* adult females, we first extract the 3D structures of all labeled neurons (1–5% of total population) for each individual brain. We then merge many partial representations by a non-linear spatial transformation into a model brain with an average size and shape. By oversampling more than five-fold of the total brain neurons, the model brain contains most if not all neuron types and all connections between local processing units.

This will be an open resource, allowing the inclusion of additional data from other laboratories. Note that in this process no assumption is made that the structure and the connections of neurons in each brain are identical after morphing, but only that when a sufficient number of specimens is reached, each neuron-to-neuron connection can be assigned a likelihood index. One can then focus on a small region of interest to validate the connection by nanoscale x-ray tomography or serial-section electron microscopy at the synapse resolution. Thus, the proposed connectome map will include all possible variations between different *Drosophila* brains and the deduced wiring diagram will guild us for further functional studies. The problem of variability between individual connectomes will be more challenging in the large brains in mice and humans.

## How can information obtained by other imaging techniques be combined with the connectome maps from x-ray imaging?

The connectome map extracted from a collection of x-ray imaging results can be improved by additional experimental data. Such data could be obtained not only with x-ray imaging but also with other techniques. As mentioned, phase contrast tomography effectively reveals certain biological structures, such as muscles and bones, without staining. Combining results from multiple techniques should not be a problem if they also preserve the in situ spatial relation between the nervous systems and the surrounding tissues. For combining structures within the brain, using well-identifiable structure landmarks and the same morph-and-fuse strategy described above, all the 3D information yielded by imaging techniques other than x-ray imaging can be registered to the corresponding correct location.

## Can laboratory-based x-ray sources provide an alternative to synchrotrons?

Modern synchrotron facilities provide unmatched performance particularly suitable for microtomography and nanotomography. However, they are large-size instruments with high operation costs; thus, their accessibility is a concern. Sources that are much smaller and have not-too-reduced performance are a key objective of x-ray technology. Miniature synchrotrons have given rather disappointing results, but other devices emitting high-brightness x-rays are becoming quite promising for high-resolution imaging.

However, their demonstrated performance—particularly the resolution—is not yet sufficient for connectome mapping. This limitation is primarily a consequence of the large source size, and could in principle be overcome with image magnification, for example, by using x-ray optics [21–23] or other solutions [24].

Even so, there remains the problem of image acquisition speed—the characteristic that makes x-ray imaging the best candidate for connectome mapping of large animal brains. This problem could be solved at least in part with the parallel use of multiple laboratory-size sources and detectors. This would also relax the requirements for specimen preparation. A parallel acquisition strategy could thus become an important part of the ultimate strategy for mapping the human connectome.

## Outlooks: what improvements are needed to eventually map human brains?

Speed and throughput still are the key concerns: every steps of the mapping process must be optimized to improve them.

- Better staining could enhance the throughput. However, this may be problematic for the whole human brain: no Golgi staining was reported for it with the quality reached on smaller scales. The solution may be a compromise between staining, sectioning, and tomography performance.
- For image acquisition, brighter x-rays from laboratory sources, synchrotrons, or even x-ray-free electron lasers would increase the throughput.
- Improvement of the detector efficiency, currently affected by technological limitations, is critical for the human-brain connectome mapping.
- Other detection improvements could boost the throughput. A projection microscopy system using an x-ray point source could scale the magnification to match the detector pixel size and get the desired resolution. This could eliminate the inefficient conversion of x-rays to visible light by scintillation, replacing the visible-light detectors with directly coupled x-ray detectors.
- A large number of small-size pixels can increase the detection area and reduce the time to slice and cut the specimens. Ideally, a detector covering the entire human brain, ~ 20 × 30 cm^2^ with a pixel size of 0.5 μm^2^, equivalent to a 60 K × 40 K camera, would allow microtomography without sectioning. This device is not yet commercially available, but feasible with the current manufacturing technologies. This, however, would imply massive computation for reconstruction, requiring a supercomputer.
- New methods for 3D x-ray imaging, including algorithms for tomography reconstruction from a small number of projection images, could lead to orders-of-magnitude improvements of the mapping throughput.
- The speed of standard computers is not optimal for tomography reconstruction, segmentation, and tracing. Supercomputers could strongly improve the throughput. Managing the resulting database will also require supercomputers linked by a very fast network.

## Should an international network for connectome mapping be created?

With the increasing rate of technological breakthroughs, a comprehensive x-ray mapping of the human connectome should become feasible in the not-too-distant future. The scale of this enterprise, however, will pose formidable challenges requiring a broad international effort. This collaboration must be structured into parallel operations, similar to the strategy implemented for the Human Genome Project.

There are more than 30 synchrotron facilities worldwide—each with tens of beamlines—suitable for microtomography and nanotomography. And the possible use of laboratory-based sources would result in an even broader network. This, by itself, speaks in favor of international cooperation.

Thus, the planning of the connectome mapping strategy must include the effective management of a large international consortium. Its individual nodes must be organized to perform their cooperative tasks in parallel first, and afterwards to focus on specific applications, taking advantage of the combined knowledge generated by the entire collaboration. The Human Brain Project [25] or Graphene [26] of Future and Emerging Technology Flagship Initiatives in Europe provides relevant experiences about the strong points but also about problems: they should constitute the references for the envisioned X-ray Connectome Consortium.

## References

[1] Hwu Y, Tsai WL, Chang HM, Yeh HI, Hsu PC, Yang YC (2004). Imaging cells in tissues with refractive index radiology. Biophys J.

[2] Margaritondo G (2002). Elements of synchrotron light: for biology, chemistry and medical research.

[3] Chien CC, Tseng PY, Chen HH, Hua TE, Chen ST, Chen YY (2013). Imaging cells and sub-cellular structures with ultrahigh resolution full-field X-ray microscopy. Biotechnol Adv.

[4] Chen TY, Chen YT, Wang CL, Kempson IM, Lee WK, Chu YS (2011). Full-field microimaging with 8 keV X-rays achieves a spatial resolutions better than 20 nm. Opt Express.

[5] Wu SR, Hwu Y, Margaritondo G (2012). Hard-X-ray zone plates: recent progress. Materials.

[6] Wu HR, Chen ST, Chu YS, Conley R, Bouet N, Chien CC (2012). Nanoresolution radiology of neurons. J Phys D.

[7] Buzug TM (2008). Computed tomography: from photon statistics to modern cone-beam CT.

[8] Margaritondo G, Hwu Y, Je JH (2004). Synchrotron light in medical and materials science radiology. Riv Novo Cimento.

[9] Hwu Y, Tsai WL, Je JH, Seol SK, Kim B, Groso A (2004). Synchrotron microangiography with no contrast agent. Phys Med Biol.

[10] Cecilia A, Rack A, Doussard PA, Martin T, dos Santos Rolo T, Vagovic P (2011). LPE grown LSO:Tb scintillator films for high-resolution X-ray imaging applications at synchrotron light sources. Nucl Instr Meth Phys Res A.

[11] Pasternak JF, Woolsey TA (1975). On the “selectivity” of the Golgi-Cox method. J Comp Neur.

[12] Li A, Gong H, Zhang B, Wang Q, Yan C, Wu J, Liu Q, Zeng S, Luo Q (2010). Micro-optical sectioning tomography to obtain a high-resolution atlas of the mouse brain. Science.

[13] Kalender WA (2011). Computed tomography: fundamentals, system technology, image quality, applications.

[14] Chien CC, Kempson IM, Wang CL, Chen HS, Hwu Y, Chen NY (2013). Complete microscale profiling of tumor microangiogenesis: a microradiological methodology reveals fundamental aspects of tumor angiogenesis and yields an array of quantitative parameters for its characterization. Biotechnol Adv.

[15] Calhoun PS, Kuszyk BS, Heath DG, Carley JC, Fishman EK (1999). Three-dimensional volume rendering of spiral CT data: theory and method. RadioGraphics.

[16] Mei C, Sibley G, Cummins M, Newman P, Reid I (2011). REAL: a system for large-scale mapping in constant-time using stereo. Int J Comput Vision.

[17] Markram H, Muller E, Ramaswamy S, Reimann MW, Abdellah M, Sanchez CA (2015). Reconstruction and simulation of neocortical microcircuitry. Cell.

[18] He GW, Wang TY, Chiang AS, Ching YT. Soma detection in 3D images of neurons using machine learning technique. Neuroinformatics. 2017;doi: 10.1007/s12021-017-9342-0. 10.1007/s12021-017-9342-0 29032511

[19] Chiang AS, Lin CY, Chuang CC, Chang HM, Hsieh CH, Yeh CW (2011). Three-dimensional reconstruction of brain-wide wiring networks in drosophila at single-cell resolution. Curr Biol.

[20] Shih CT, Sporns O, Yuan SL, Su TS, Lin YJ, Chuang CC (2015). Connectomics-based analysis of information flow in the *Drosophila* brain. Curr Biol.

[21] Mimura H, Handa S, Kimura T, Yumoto H, Yamakawa D, Yokoyama H (2010). Breaking the 10 nm barrier in hard-X-ray focusing. Nat Phys.

[22] Matsuyama S, Nakamori H, Goto T, Kimura T, Khakurel KP, Kohmura Y (2016). Nearly diffraction-limited X-ray focusing with variable-numerical-aperture focusing optical system based on four deformable mirrors. Sci Rep.

[23] Nazaretski E, Xu W, Bouet N, Zhou J, Yan H, Huang X, Chu Y (2016). Development and characterization of monolithic multilayer Laue lens nanofocusing optics. Appl Phys Lett.

[24] Cosslett VE, Nixon WC (1952). An experimental X-ray shadow microscope. Proc R Soc.

[25] Human Brain Project. https://www.humanbrainproject.eu/. Accessed 29 Nov 2017.

[26] Graphene Flagship. https://www.graphene-flagship.eu/. Accessed 29 Nov 2017.

